# Investigation on Mercury Reemission from Limestone-Gypsum Wet Flue Gas Desulfurization Slurry

**DOI:** 10.1155/2014/581724

**Published:** 2014-03-04

**Authors:** Chuanmin Chen, Songtao Liu, Yang Gao, Yongchao Liu

**Affiliations:** School of Environmental Science & Engineering, North China Electric Power University, Baoding 071003, China

## Abstract

Secondary atmospheric pollutions may result from wet flue gas desulfurization (WFGD) systems caused by the reduction of Hg^2+^ to Hg^0^ and lead to a damping of the cobenefit mercury removal efficiency by WFGD systems. The experiment on Hg^0^ reemission from limestone-gypsum WFGD slurry was carried out by changing the operating conditions such as the pH, temperature, Cl^−^ concentrations, and oxygen concentrations. The partitioning behavior of mercury in the solid and liquid byproducts was also discussed. The experimental results indicated that the Hg^0^ reemission rate from WFGD slurry increased as the operational temperatures and pH values increased. The Hg^0^ reemission rates decreased as the O_2_ concentration of flue gas and Cl^−^ concentration of WFGD slurry increased. The concentrations of O_2_ in flue gas have an evident effect on the mercury retention in the solid byproducts. The temperature and Cl^−^ concentration have a slight effect on the mercury partitioning in the byproducts. No evident relation was found between mercury retention in the solid byproducts and the pH. The present findings could be valuable for industrial application of characterizing and optimizing mercury control in wet FGD systems.

## 1. Introduction

Mercury and its compounds are highly toxic species which have a considerable impact on human health. A large proportion of mercury is emitted to the environment by the burning of coal. This process is responsible for about one-third of anthropogenic mercury emissions [1, 2]. Mercury may be present in flue gas as elemental mercury (Hg^0^) or oxidized mercury (Hg^2+^). It may also be retained in fly ash particles, in which case it is referred to as particle-bound mercury (Hg^P^). Whereas Hg^P^ is retained in the electrostatic precipitators or bag filters, both Hg^2+^ and Hg^0^ species from the flue gas are emitted to the atmosphere in power plants without undergoing any postcombustion processes to reduce emissions. In some cases, wet flue gas desulfurization (WFGD) systems installed in coal fired power plants to control SO_2_ emissions have been used to decrease mercury emissions [3–7]. In such systems, SO_2_ usually reacts with the limestone slurry to produce insoluble gypsum.

Hg^2+^ can be efficiently captured in WFGD by taking advantage of its high solubility in water [3, 5, 8]. However, the elemental mercury is difficult to capture with typical air pollution control devices (APCD) due to its volatility and chemical stability [1]. One strategy which is being explored is the use of a catalyst or oxidant to oxidize elemental mercury in the upstream of WFGD system, and then the oxidized mercury is absorbed by WFGD slurry. However, during the work aimed at enhancing the mercury-removal performance of WFGD systems, investigators discovered that a portion of absorbed oxidized mercury will be reduced to elemental mercury (Hg^0^) in WFGD system and eventually released into flue gas [9–11], and the total mercury removal efficiency was significantly limited. As such, to improve the efficiency, it is necessary to control mercury reemission from WFGD slurry to prevent from reducing the cobenefit of wet scrubber mercury removal.

Studies on elemental mercury reemission in lab- and pilot-scale WFGD systems were reported in recent years. Some researchers indicated that the reduction process presumably occurred via aqueous reduction of Hg^2+^ by sulfite ions. The process was initiated by the formation of unstable intermediate, HgSO_3_, which immediately decomposed to aqueous Hg^0^ and eventually reemitted to gas phase [11–13]. The authors also studied the effect of some operational parameters, for example, pH value, concentration of S(IV), temperature, and concentration of Cl^−^ on elemental mercury reemission [10, 14, 15]. Wo et al. [10] indicated that flue gas Hg^0^ reemission across a wet FGD scrubber can be reduced by increasing the initial pH value, concentration of S(IV), or lowering the temperature. But Wu et al. [15] had the opposite conclusion about the effect of pH on the mercury reemission. Their work suggested that Hg^0^ reemission was suppressed by decreasing the pH. They also suggested that there existed a qualitative relationship between the initial oxidation-reduction potential (ORP) values of the slurries and Hg^0^ reemission across the slurries [15]. Some literatures [10, 12] had yet concluded that the Cl^−^ had inhibition effect on the reduction of Hg^2+^, where the formation of ClHgSO_3_^−^ was suggested as the main cause for this inhibition [16].

Furthermore, these parameters not only affect Hg^0^ reemission, but also impact the partitioning behavior of mercury in the solid and liquid byproducts. To better understand the performance of Hg^0^ reemission in the wet FGD system, a sequence of experiments was carried out in order to evaluate the influence of different operational parameters on Hg^0^ reemission efficiency in a bubbling reactor and the partitioning behavior of mercury in the solid and liquid byproducts was also discussed.

## 2. Experiments and Methods

### 2.1. Experimental Apparatus

**Figure 1:**
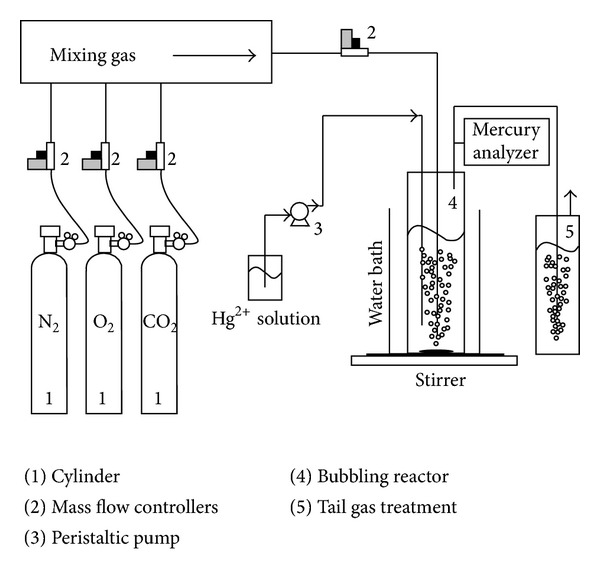
Schematic of the experiment system.

The schematic diagram of a lab-scale wet FGD simulated system is illustrated in Figure 1. The elemental mercury (Hg^0^) reemission and the factors that impact Hg^0^ reemission were investigated by using the simulated scrubber. This system consisted of an oxidized mercury (Hg^2+^) injection system, carrier gas system, scrubbing system, and mercury analyzer system. The scrubbing system was composed of a bubbling reactor, a water bath, and a magnetic stirring system. The Hg^2+^ injection system was a peristaltic pump system, which can deliver the HgCl_2_ solution to the bubbling reactor as the source as well as control and adjust its injection rates. The Hg^2+^ solution went directly to the bottom of the flask through a Teflon tube. The carrier gas system included cylinder gases, mass flow controllers (MFCs), and delivery piping, which was made of Teflon tubes. The desired flow rates of the carrier gases were controlled by calibrated MFCs. The reaction solution was stirred under N_2_, O_2_, and CO_2_ atmosphere to remove the produced Hg^0^.

### 2.2. Experimental Procedure

At the beginning of each test, a slurry with the desired concentration (1% w/w) was prepared and poured into the reactor; the reactor was submerged into the water bath at the desired temperature. The CaSO_4_ and CaSO_3_ (mixing rate, 90/10) were used to simulate the slurry of the limestone-forced oxidation wet FGD system. The Hg^2+^ injection system was a peristaltic pump system, which can deliver the HgCl_2_ solution to the bubbling reactor as the source of Hg^2+^ as well as control and adjust its injection rates. A 50 *μ*g/l Hg^2+^ solution was pumped into the reactor at a rate of 10 mL/h. The initial pH of the solution was controlled through the combined addition of CaCO_3_ and H_2_SO_4_ to the reactor and measured by pH meter. Other chemicals, such as NaCl as the source of Cl^−^, were selectively added to the bubbling reactor. The carrier gas with a flow rate of 1000 mL/min was introduced into the scrubber. The carrier gas came in contact with the slurry through the scrubber. Then, the carrier gas arrived at the mercury analyzer, which initiated the test. When the blank testing values of the mercury concentrations in the carrier gas were stable, the Hg^2+^ solution was injected. Continuous Hg^0^ concentration detection at the outlet of the simulated WFGD reactor was started at this point by a LUMEX RA-915+ Hg analyzer until the steady state was achieved. The mercury content of the solid and aqueous samples generated in the lab-scale tests was also determined by means of LUMEX RA-915+ Hg analyzer. No oxidized mercury was detected through multiple tests because the oxidized mercury dissolved in the slurry. The elemental mercury concentration that was emitted from the slurry was tested to quantify the elemental mercury reemission levels. The mercury concentrations were recorded once per minute. The mercury mass balance for each test was calculated. It is found that the error of the overall mercury mass balance was in the range of 94%–105% for all tests. The range of experimental conditions used for the scrubber slurry and the simulated flue gas is included in Table 1.

### 2.3. Mercury Reemission Efficiency Calculation

In this paper, mercury reemission efficiency (*η*_Hg^0^_) was calculated by the equation listed as follows:

(1) $$\begin{array}{c} \eta_{\text{H}{\text{g}}^{0}}=\frac{c_{{\text{Hg}}_{\text{out}}^{0}}}{c_{\text{H}{\text{g}}_{\text{in}}^{2+}}}\times 100\%, \end{array}$$

where *η*_Hg^0^_ is the mercury reemission efficiency, *c*_Hg_in_^2+^_ is the inlet Hg^2+^ concentrations, and *c*_Hg_out_^0^_ is the outlet Hg^0^ concentration.

## 3. Results and Discussion

### 3.1. Effect of the Oxygen Concentration in the Flue Gas on Hg^0^ Reemission

**Figure 2:**
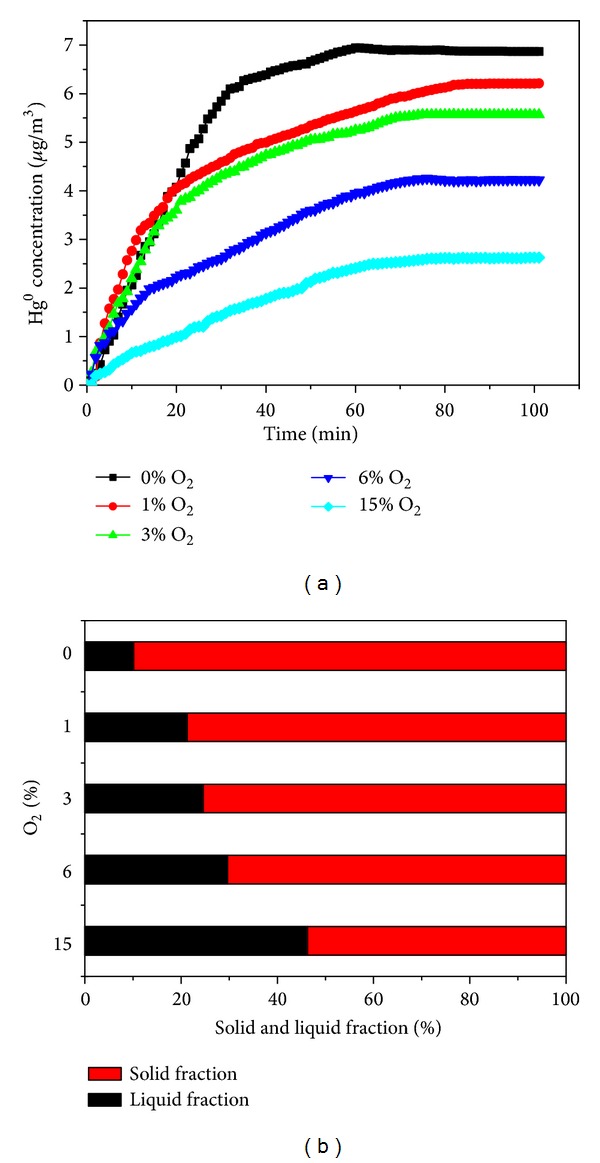
(a) Effect of the oxygen concentration in the flue gas on Hg^0^ reemission. (b) Relationship between the proportion of mercury retained in the solid and liquid fraction of the slurry and the concentration of oxygen concentration in the flue gas.

The impact of oxygen concentration in the flue gas on Hg^0^ reemission from the simulated WFGD slurry is shown in Figure 2. The experiments reported in Figure 2 were performed at a pH of 5.5 and a temperature of 55°C. The experimental range of oxygen concentration used in these experiments was from 0% to 15%. From Figure 2(a), it can be seen that the Hg^0^ reemission rates increase as the oxygen concentration in the flue gas increases. The Hg^0^ concentration in flue gas reached about 6.87 *μ*g/m^3^ for 0% O_2_ at 100 min when Hg^0^ concentration was stable. In contrast, only 2.62 *μ*g/m^3^ was obtained for 15% O_2_. The Hg^0^ reemission reaction mechanism is explained by using the chemical reaction in [15]

(2) $$\begin{array}{c} {\text{Hg}}^{2+}+{\text{HS}{\text{O}}_{3}}^{-}+{\text{H}}_{2}\text{O}⟷{\text{Hg}}^{0}+{\text{S}{\text{O}}_{4}}^{2-}+3{\text{H}}^{+} \end{array}$$

The SO_3_^2−^ was oxidized into SO_4_^2−^ through reaction (3) when the carrier gas that contained O_2_ was blown into the scrubber. Thus, the concentration of HSO_3_^−^ was decreased, which resulted in a lower Hg^0^ reemission rate:

(3) $$\begin{array}{c} 2{\text{S}{\text{O}}_{3}}^{2-}+{\text{O}}_{2}⟶2{\text{S}{\text{O}}_{4}}^{2-} \end{array}$$

The mercury partitioning in the byproducts indicates that an increase in mercury retention in the solid fraction occurs at lower concentrations of O_2_ in flue gas (Figure 2(b)). This suggests that sulfate ions may be contributing to the formation of a small amount of mercury sulfate which then precipitates with the gypsum particles or decomposes in HgO(s) [14].

### 3.2. Effect of the Temperature on Hg^0^ Reemission

**Figure 3:**
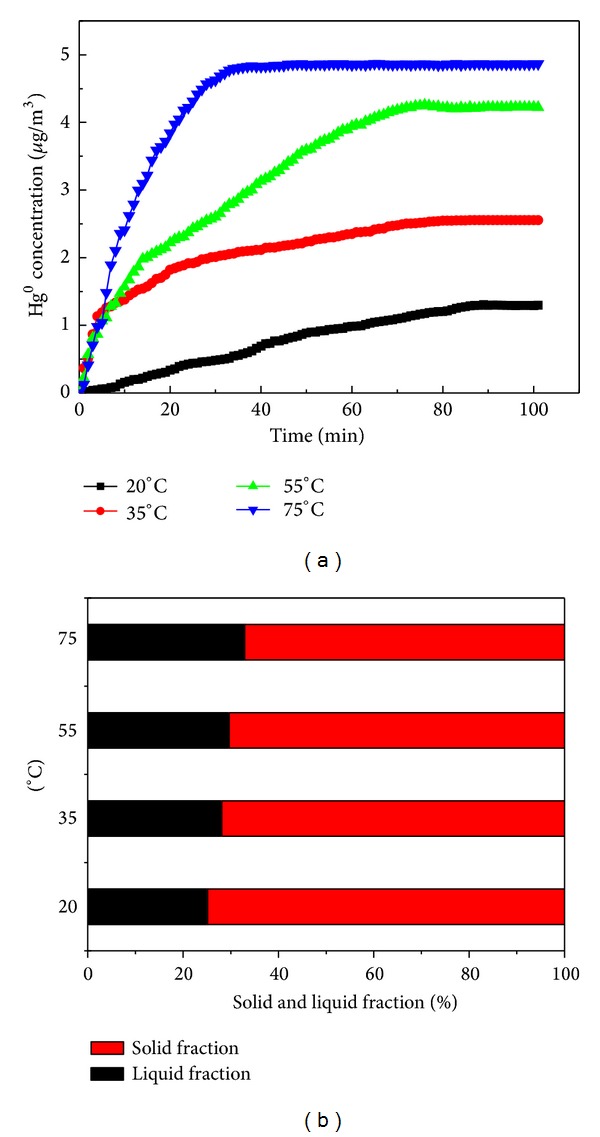
(a) Effect of the temperature on Hg^0^ reemission. (b) Relationship between the proportion of mercury retained in the solid and liquid fraction of the slurry and the temperature.

The tests of Hg^0^ reemission at different temperatures were conducted. The experimental range of temperature used in these experiments was from 20 to 75°C by adjusting the temperature of the water bath. The pH value of the slurry was kept at 5.5.  Figure 3(a) shows the elemental mercury concentrations versus the HgCl_2_ injection time at four temperature levels. It can be seen that the Hg^0^ reemission rate increases with the temperature of the simulated scrubber. The Hg^0^ concentration in flue gas reached about 4.87 *μ*g/m^3^ at 75°C at an injection time of 100 min, while it was only 1.29 *μ*g/m^3^ at 20°C.  Figure 3(b) shows that there was a slight decrease in mercury retention in the solid fraction with the temperature rising.

### 3.3. Effect of the pH on Hg^0^ Reemission

**Figure 4:**
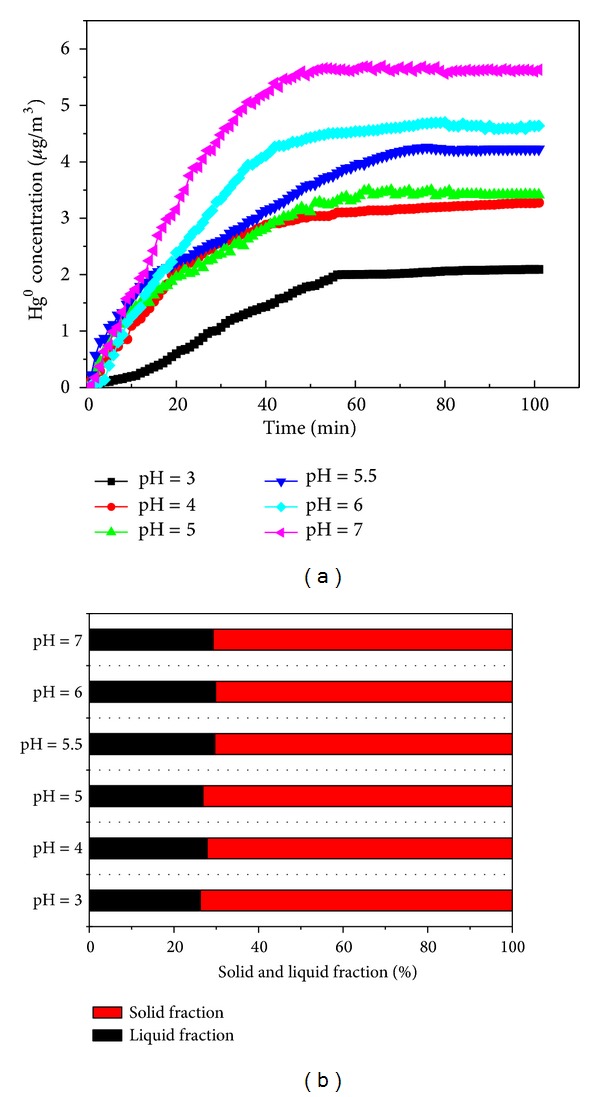
(a) Effect of the pH on Hg^0^ reemission. (b) Relationship between the proportion of mercury retained in the solid and liquid fraction of the slurry and the pH.

Six tests at different initial pH values (3, 4, 5, 5.5, 6, and 7) were conducted in the simulated scrubber. The temperature of the solution was 55°C. The Hg^0^ concentration curves at different pH values are shown in Figure 4. From Figure 4(a), it can be found that the Hg^0^ reemission rates increase as the pH values increase. The Hg^0^ concentration in flue gas reached about 5.60 *μ*g/m^3^ for pH = 7 at 100 min. In contrast, only 2.09 *μ*g/m^3^ was obtained for pH = 3. Equation (2) was a reversible reaction, according to the principle of chemical reactions, and a counter reaction was performed at a lower pH value, where the concentration of H^+^ was high. Therefore, the Hg^0^ reemission rate decreased in the solution as the pH value decreased. From Figure 4(a), it can be seen that the pH seems to have no effect on the mercury partitioning in the byproducts.

### 3.4. Effect of the Cl^−^ Concentration on Hg^0^ Reemission

**Figure 5:**
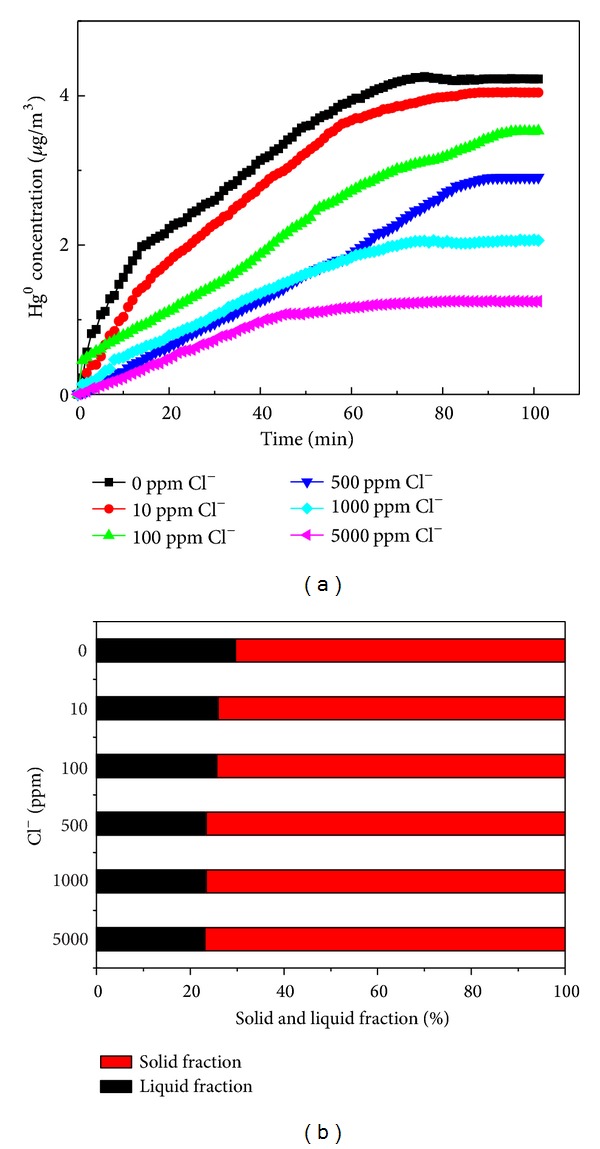
(a) Effect of the Cl^−^ concentration on Hg^0^ reemission. (b) Relationship between the proportion of mercury retained in the solid and liquid fraction of the slurry and the Cl^−^.

Figure 5 presents the effect of Cl^−^ concentration on Hg^0^ reemission. The experiments were carried out at the pH value of 5.5 and the temperature of 55°C. It can be seen that Cl^−^ concentration has an evident effect on the Hg^0^ reemission. The Hg^0^ reemission shows that the fastest reaction rate in the simulated desulfurization slurry is without chloride and the Hg^0^ reemission rate decreases with Cl^−^ increasing. From Figure 5 it can be seen that the Hg^0^ concentration in flue gas reached about 4.23 *μ*g/m^3^ without Cl^−^ at 100 min while only 1.24 *μ*g/m^3^ with 5000 ppm Cl^−^.

As is found, the reactions for Hg^0^ emission are as follows: the main pathway is through mercuric-sulfite complexes [11, 13]:

(4) $$\begin{array}{c} {\text{Hg}}^{2+}+{\text{S}{\text{O}}_{3}}^{2-}⟷\text{HgS}{\text{O}}_{3} \end{array}$$

(5) $$\begin{array}{c} \text{HgS}{\text{O}}_{3}+{\text{S}{\text{O}}_{3}}^{2-}⟷\text{Hg}{\left({\text{S}{\text{O}}_{3}}^{2-}\right)}_{2} \end{array}$$

(6) $$\begin{array}{c} \text{HgS}{\text{O}}_{3}+{\text{H}}_{2}\text{O}⟶{\text{Hg}}^{0}↑+{\text{S}{\text{O}}_{4}}^{2-}+2{\text{H}}^{+} \end{array}$$

(7) $$\begin{array}{c} \text{Hg}{\left({\text{S}{\text{O}}_{3}}^{2-}\right)}_{2}+{\text{H}}_{2}\text{O}⟶{\text{Hg}}^{0}↑+2{\text{S}{\text{O}}_{4}}^{2-}+2{\text{H}}^{+} \end{array}$$

New mercuric-sulfite-chloride complexes ClHgSO_3_^−^ and Cl_2_HgSO_3_^2−^ are formed through the following reactions when the chloride is added into the simulated desulfurization solutions:

(8) $$\begin{array}{c} \text{HgS}{\text{O}}_{3}+{\text{Cl}}^{-}⟷\text{ClHg}{\text{S}{\text{O}}_{3}}^{-} \end{array}$$

(9) $$\begin{array}{c} \text{ClHg}{\text{S}{\text{O}}_{3}}^{-}+{\text{Cl}}^{-}⟷{\text{Cl}}_{2}\text{Hg}{\text{S}{\text{O}}_{3}}^{2-} \end{array}$$

ClHgSO_3_^−^ can decompose to Hg^0^ through the reaction (10). But the decomposition rate of ClHgSO_3_^−^ is much slower than HgSO_3_ or Hg(SO_3_^2−^)_2_ (reactions (6) and (7)). In addition, Cl_2_HgSO_3_^2−^ is formed reversibly at higher chloride concentration, which does not decompose to Hg^0^ [12, 16]:

(10) $$\begin{array}{c} \text{ClHg}{\text{S}{\text{O}}_{3}}^{-}+{\text{H}}_{2}\text{O}⟷\;{\text{Hg}}^{0}↑+{\text{S}{\text{O}}_{4}}^{2-}+{\text{Cl}}^{-}+2{\text{H}}^{+} \end{array}$$

From Figure 5(b), it can be seen that the Cl^−^ seems to have a slight effect on the mercury partitioning in the byproducts. The proportion of mercury retained in the solid decreases from 76.85% to 70.31% when the Cl^−^ concentration in the slurry increases from 0 ppm to 5000 ppm.

## 4. Conclusions

An evaluation of the influence of the operating conditions, which included the pH, temperature, Cl^−^ concentrations, and oxygen concentrations, on Hg^0^ reemission from wet flue gas desulfurization slurry was carried out. The experimental results indicated that the Hg^0^ reemission rate from WFGD slurry increased as the operational temperatures and pH values increased. However, the Hg^0^ reemission rates decreased as the O_2_ concentration of flue gas and Cl^−^ concentration of WFGD slurry increased. So the Hg^0^ reemission from WFGD system can be reduced or slowed by decreasing the temperature and pH or by using forced oxidation. The results of mercury partitioning behavior in the solid and liquid byproducts show that mercury retention in the solid fraction increased with the concentrations of O_2_ in flue gas decreasing and slightly decreased in mercury retention in the solid fraction with the temperature and Cl^−^ concentration in the slurry rising. And there is no evident relation between mercury retention in the solid byproducts and the pH. The present findings could be valuable for industrial application of characterizing and optimizing mercury control in wet FGD systems.

## Figures and Tables

**Table 1:** Experimental conditions.

Parameter	Simulated WFGD slurry	Simulated flue gas
Reagent	90%CaSO_4_/10%CaSO_3_	—
Initial pH	3–7	—
Temperature (°C)	20–75	20
O_2_ (vol.%)	—	0–15
CO_2_ (vol.%)	—	12
N_2_ (vol.%)	—	As balance
Gas flow rate (mL/min)	—	1000
Hg^2+^ concentration (*μ*g/L)	50	—
Hg^2+^ injection rate (mL/h)	10	—
Hg^2+^ concentration at scrubbing system inlet (calculated in gas, *μ*g/m^3^ gas)	—	8.3
Cl^−^ (ppm)	0–5000	—
